# Editorial: Secondary metabolites as multifunctional molecules in the changing environment of plant growth

**DOI:** 10.3389/fpls.2023.1266602

**Published:** 2023-08-22

**Authors:** Maciej Strzemski, Agnieszka Hanaka

**Affiliations:** ^1^ Department of Analytical Chemistry, Faculty of Pharmacy, Medical University of Lublin, Lublin, Poland; ^2^ Department of Plant Physiology and Biophysics, Institute of Biological Sciences, Faculty of Biology and Biotechnology, Maria Curie-Skłodowska University, Lublin, Poland

**Keywords:** secondary metabolites, plant physiology, biotic and abiotic stresses, agricultural and pharmaceutical sciences, metabolomics

Metabolites derived from specialized plant metabolism, known as secondary metabolites, serve various important functions in plant organisms. Many of these compounds have already been identified and fairly well described. Secondary metabolites play a significant role in minimizing oxidative stress caused by chemical and physical agents by increasing the biosynthesis of compounds with antioxidant activity (Dresler et al., 2020). They also contribute to the synthesis of substances with pesticidal properties in response to plant pathogen infections or mechanical damage caused by insects to plant tissues. Furthermore, these compounds interact with other plant organisms and microorganisms, acting as attractants for pollinating insects or insects that parasitize plant pests (Oleszek et al., 2001). Additionally, secondary metabolites can be integral components of the cell’s liquid matrix, with molecular structures resembling deeply eutectic solvents (Choi et al., 2011; Verpoorte et al., 2022).

Given that living organisms do not expend energy unnecessarily and considering the vast number of secondary metabolites identified in plant tissues, it can be assumed that each compound serves a specific role. In this context, our understanding of the importance of secondary metabolites in plant life appears to be in its early stages and warrants further intensive research. Acquisition of this knowledge is crucial not only for enhancing our understanding of plant physiology and ecology but also for its practical value in the development of agricultural and pharmaceutical sciences. Many secondary metabolites exhibit potential as effective pesticides (Spinozzi et al., 2023), plant growth stimulants (Oleszek et al., 2001), or medicinal drugs (Verpoorte, 2017). Understanding the influence of various chemical and physical factors on the biosynthesis of secondary metabolites may facilitate their efficient production in field crops and *in vitro* cultures, ultimately having an impact on the quality of yields.

This Research Topic encompasses five original research articles (Grzelka et al.; Gašić et al.; Liu et al.; Li et al.; Yang et al.). The articles compiled here reflect the extensive scope of the role of plant secondary metabolites in the plant growth environment and can serve as inspiration for further research endeavors.

Grzelka et al. studied the effect of electroporation on the growth of *Scuttelaria baicalensis* plants and on the content of flavonoids (especially baicalein and wogonin) in the pharmacognostic raw material obtained with this method. The authors succeeded in obtaining plants containing more than twice as many flavonoids as control plants. Thus, they seem to have pointed the way to the acquisition of high-quality raw material from this medicinally important plant.

Gašić et al. conducted a detailed study of the plant metabolome of three species of the genus *Digitalis*. The plants came from several dozen sites located in Serbia and Bosnia and Herzegovina. The authors identified 115 metabolites, indicating species-specific compounds. They also conducted a quantitative analysis of sixteen metabolites and, using advanced methods of numerical data analysis, demonstrated the relatively species-specific content of each metabolite, with no significant effect of the environmental conditions and geographic location on the metabolite profile.

Liu et al. isolated four new phenylpropanoid amides from the invasive plant *Solanum rostratum* and demonstrated the outstanding phytotoxic activity of these compounds against *Arabidopsis thaliana*. This interdisciplinary work conducted using modern methods of chemical instrumental analysis and *in vitro*, *in vivo*, and *in silico* biological studies significantly expands the knowledge of plant-plant interactions and succession mechanisms and may have great application value, being part of the trend towards the search for new natural herbicides.

Li et al. investigated the effect of hetero-grafting on the biosynthesis and accumulation of flavonoids in sweet orange fruit. They showed that the type of rootstocks used has a decisive influence on the level of expression of genes regulating the biosynthesis of flavonoids and, as a result, on the content of flavonoids. This study not only expands our understanding of flavonoid biosynthesis, but may also have a direct impact on the methodology of orange cultivation.

Yang et al. studied the effect of exogenous abscisic acid on the ripening and chemical composition of *Vaccinium corymbosum* fruits. They showed that spraying with abscisic acid at a concentration of 1000mg/l caused fruit to ripen faster and had a positive effect on the content of anthocyanins and endogenous abscisic acid. Despite the seemingly exclusively application-related objective of the research, the authors conducted detailed mechanistic studies aimed at elucidation of the observed changes, thus expanding the knowledge in the field of physiology and biochemistry of *Vaccinium corymbosum*.

The articles published in this Research Topic therefore cover a wide spectrum of issues related to phytochemistry and plant physiology. All published studies were of application nature with a simultaneous intention to understand the observed effects at the molecular level. This ambivalent nature of the research, combined with interdisciplinarity and the use of the most modern research techniques and methods, allows us to believe that the authors contribute to pushing the frontiers in plant research.

## Author contributions

MS: Conceptualization, Project administration, Writing – original draft, Writing – review & editing. AH: Conceptualization, Project administration, Writing – original draft, Writing – review & editing.
